# Pericyte Structural Remodeling in Cerebrovascular Health and Homeostasis

**DOI:** 10.3389/fnagi.2018.00210

**Published:** 2018-07-17

**Authors:** Andrée-Anne Berthiaume, David A. Hartmann, Mark W. Majesky, Narayan R. Bhat, Andy Y. Shih

**Affiliations:** ^1^Department of Neuroscience, Medical University of South Carolina, Charleston, SC, United States; ^2^Center for Developmental Biology and Regenerative Medicine, Seattle Children’s Research Institute, Seattle, WA, United States; ^3^Departments of Pediatrics and Pathology, University of Washington, Seattle, WA, United States; ^4^Center for Biomedical Imaging, Medical University of South Carolina, Charleston, SC, United States

**Keywords:** pericyte, two-photon imaging, capillary blood flow, blood-brain barrier, Alzheimer’s disease, mural cell, stroke, neurovascular coupling

## Abstract

The biology of brain microvascular pericytes is an active area of research and discovery, as their interaction with the endothelium is critical for multiple aspects of cerebrovascular function. There is growing evidence that pericyte loss or dysfunction is involved in the pathogenesis of Alzheimer’s disease, vascular dementia, ischemic stroke and brain injury. However, strategies to mitigate or compensate for this loss remain limited. In this review, we highlight a novel finding that pericytes in the adult brain are structurally dynamic *in vivo*, and actively compensate for loss of endothelial coverage by extending their far-reaching processes to maintain contact with regions of exposed endothelium. Structural remodeling of pericytes may present an opportunity to foster pericyte-endothelial communication in the adult brain and should be explored as a potential means to counteract pericyte loss in dementia and cerebrovascular disease. We discuss the pathophysiological consequences of pericyte loss on capillary function, and the biochemical pathways that may control pericyte remodeling. We also offer guidance for observing pericytes *in vivo*, such that pericyte structural remodeling can be more broadly studied in mouse models of cerebrovascular disease.

## Introduction

Brain pericytes nurture the development and maintenance of a healthy cerebrovascular system. In concert with endothelial cells, they support numerous vascular functions including blood-brain barrier (BBB) integrity, cerebral blood flow regulation, vessel stability, angiogenesis and immune cell trafficking (Sweeney et al., 2016). Pericytes have also been suggested as a source of adult multipotent stem cells (Dore-Duffy, 2008; Nakagomi et al., 2015), although whether this attribute persists *in vivo* was recently questioned (Guimarães-Camboa et al., 2017). The coordination of molecular signaling between pericytes and endothelial cells is crucial for a properly organized microvascular network, a subject which has been extensively studied in the context of brain development (Armulik et al., 2005, 2011). However, less is known about pericyte-endothelial communication in the adult brain and the implications of disrupted signaling in age-related cerebrovascular diseases such as dementia and stroke.

The purpose of this review is to describe “pericyte structural remodeling,” a novel facet of pericyte biology we have recently observed in the adult mouse brain using long-term high-resolution optical imaging (Berthiaume et al., 2018). Pericyte structural remodeling is the adaptive extension of pericyte processes along microvessel walls on the time-scale of days. It is an under-explored mechanism by which pericytes react on-demand to preserve endothelial contact in the adult brain. As a prelude to further discussion of this topic, we briefly describe the importance of pericyte-endothelial signaling during vascular development and maturation, followed by an account of the current knowledge on pericyte changes during aging. Specifically, we emphasize the vulnerability of pericytes in age-related cerebrovascular and neurodegenerative diseases, particularly Alzheimer’s disease. Next, we focus on the structural remodeling of pericytes that we have visualized using *in vivo* two-photon imaging of the adult mouse cortex. This includes an updated view of the identity and topography of pericytes and smooth muscle cells (SMCs) in the adult brain vasculature, which is an evolving topic in the cerebrovascular field. Finally, we discuss potential mechanisms underlying the control of pericyte structural remodeling, which may be targeted for experimental exploration or potential therapeutic benefit in the adult or aging brain. We would like to emphasize that the phenomenon discussed in this article refers specifically to the dynamic remodeling of pericyte shape and size rather than the oft-discussed phenotypic (cell fate) or functional plasticity of these cells (Lange et al., 2013; Holm et al., 2018).

## Pericyte-Endothelial Dynamics From Development to Maturation

How is a capillary bed established in the developing brain? The complexity of this task cannot be understated, as it involves the coordinated layering of different vascular cell types and structures to establish critical properties, such as BBB integrity and the microvascular tone needed to regulate blood flow in a limited cranial space. It further involves a delicate balance between angiogenesis and microvascular pruning to shape the angioarchitecture in a way that ensures nutrient supply to all brain cells. At the heart of this immense task is the dynamic interplay between pericytes and endothelial cells, which lays the foundation for the microvascular network.

Multiple pericyte-endothelial signaling pathways are involved in the transition of dynamic, growing vasculature in the developing brain to stable, mature networks of the adult brain. These pathways have been described in detail by excellent reviews (Armulik et al., 2005), and are only selectively discussed here as a preamble to the topic of pericyte structural remodeling. During angiogenesis, endothelial cells proliferate and migrate to form a nascent capillary tube. At the end of each new vascular stalk is an endothelial tip cell that guides vessel growth, stimulated by the release of vascular endothelial growth factor (VEGF; Gerhardt et al., 2003). New endothelial tubes are permeable and unstable until covered by pericytes. A key step in coordinating this coverage is an increase in PDGF-B/PDGFR-β signaling, which promotes the co-migration of pericytes (or pericyte precursors) to populate nascent capillaries and offsets the expression of VEGF (Hellström et al., 1999). The growth factor PDGF-B is secreted by endothelial cells, dimerizes to form PDGF-BB, and binds to the vascular extracellular matrix. PDGF-BB is sensed by pericytes, which express PDGFR-β, initiating pericyte proliferation and migration. Recruited pericytes promote the growth arrest and survival of endothelial cells, partially through TGFβ signaling (Goumans et al., 2002; Walshe et al., 2009), and by doing so, ensure vessel stability and homeostasis. While difficult to observe in developing mammals, the highly dynamic co-migration of pericytes and endothelial cells was recently captured by elegant live imaging studies using *ex utero* developing zebrafish (Ando et al., 2016). Pericytes appeared to “crawl” along endothelial tubes with peristaltic activity involving extension and contraction of long processes extending from the cell soma.

The importance of pericyte-endothelial signaling to cerebrovascular health is underscored in seminal studies involving congenital deletion of platelet-derived growth factor receptor-β (PDGFR-β) or its ligand PDGF-B. Deletion of either receptor or ligand is embryonic lethal, and the microvasculature develops aberrantly because of an inability to recruit pericytes to the endothelium (Lindahl et al., 1997). The resulting dearth of pericyte coverage is associated with endothelial hyperplasia, increased lumen diameters, and greater vascular permeability than wild-type mice (Hellström et al., 2001). Subsequent studies have used mice with varying levels of PDGF-B/PDGFR-β deficiency to show that the degree of pericyte coverage is strongly correlated with capillary BBB integrity in both developing and adult brains (Armulik et al., 2010; Daneman et al., 2010). Pericytes influence endothelial permeability by suppressing the formation of endothelial caveolae as well as the expression of leukocyte adhesion molecules (Armulik et al., 2010; Daneman et al., 2010; Ben-Zvi et al., 2014). They further influence astrocytes by modifying the polarization of their end-feet along the abluminal side of the capillary wall (Armulik et al., 2010).

Another pathway relevant to our topic is Eph-ephrin signaling, which plays a key role in tissue patterning processes during developmental morphogenesis (Kania and Klein, 2016). Eph receptor tyrosine kinases and their membrane bound ligands, called ephrins, are involved in short-range cell-cell communication. These are well-characterized as a source of cell repulsion and adhesion signaling in axon guidance, which continue to be relevant for the maintenance of cell-to-cell boundaries in adulthood (Yamaguchi and Pasquale, 2004; Evans et al., 2007). Interestingly, there also appears to be a role for these bidirectional signaling partners in blood vessel assembly (Wang et al., 1998; Salvucci et al., 2009). Though better understood as a determinant of endothelial cell arteriovenous identity, mural cell-specific consequences of impaired Eph-ephrin signaling was demonstrated by genetic deletion of a floxed *Efnb2* allele (encoding the Eph receptor ligand Ephrin B2) in mural cells of the dermal vasculature (Foo et al., 2006). This deletion did not affect mural cell numbers but resulted in pericytes and SMCs that were loosely attached to the endothelium, rounded in morphology, and improperly spread along the vasculature. Thus, EphB/ephrin-B2 signaling is required for the proper integration and organization of pericytes along the microvasculature following their recruitment. However, whether this type of signaling is as influential in the context of adult central nervous system microvasculature has yet to be determined.

As the vasculature matures in the healthy brain, the presence of pericytes continues to promote endothelial cell quiescence and vessel stabilization. The sustained activation of endothelial cell Tie2 receptors by mural cell-expressed ligand angiopoietin 1 (Ang1) contributes to the maintenance of a stable, non-leaky endothelium throughout adulthood (Augustin et al., 2009). Furthermore, the vascular basement membrane, which pericytes help to generate (Stratman et al., 2009), completely surrounds the pericytes and adds to the barrier properties of the vascular wall (Gautam et al., 2016). Direct pericyte-endothelial contact is made through the basement membrane at peg-and-socket junctions, which are inter-digitations of the pericyte and endothelial membranes bound by adhesion molecules, notably N-cadherin. The adhesion of pericytes and upregulation and translocation of endothelial N-cadherin involves a variety of signaling pathways, including TGF-β, Notch and sphingosine-1-phosphate signaling (Armulik et al., 2011). Consistent with a stabilized microvascular system in the adult brain, long-term imaging of adult mouse cerebral cortex has shown only rare formations and eliminations of capillary branches (Lam et al., 2010). This stability is further extended to pericytes, as studies from our lab, and others, have shown that pericyte cell bodies are immobile over months of imaging (Cudmore et al., 2017; Berthiaume et al., 2018). Thus, pericytes migrate to vessels during development, but are fixed in place in healthy adult brain capillaries.

## Pericyte Pathology in Aging and Age-Related Brain Diseases

Recently, there has been a strong push to understand how dysfunction of small brain vessels contributes to cognitive decline within the context of cerebrovascular and neurodegenerative disease (Snyder et al., 2015; Corriveau et al., 2016). Studies suggest that the BBB deteriorates progressively in the aging brain due to structural, cellular and molecular deficits in the neurovascular unit. These abnormalities include endothelial atrophy, thickening and irregularity of basement membranes, microvessel thinning (string vessels), increased capillary tortuosity, as well as capillary rarefaction and degeneration (Brown and Thore, 2011; Hunter et al., 2012; Bhat, 2015). With regard to pericyte pathology, a decrease in pericyte numbers has been reported with aging in both human and preclinical models, as well as ultrastructural changes, suggestive of pericyte degeneration (Erdő et al., 2017). Consistent with these observations, a recent transcriptomic and histological study of neurovasculature in the aged mouse brain (18–24 months) showed significant pericyte loss, reduced basement membrane integrity and increased endothelial transcytosis (Soto et al., 2015). Moreover, pericyte-deficient genetic mouse lines with less severe phenotypes often survive to adulthood, and have been useful in implicating pericyte loss as an accelerator of age-dependent BBB breakdown and cerebral hypoperfusion, which precede neurodegeneration and cognitive impairment (Bell et al., 2010; Kisler et al., 2017).

Pericyte status in the aging human brain has also been examined in relation to BBB function using dynamic contrast-enhanced MRI, revealing that an early degeneration of pericytes (as determined by pericyte markers in the cerebral spinal fluid) correlates with increased BBB permeability within the hippocampus (Montagne et al., 2015). Further, *post-mortem* analyses in a number of recent studies have confirmed loss of pericyte number and coverage in the cortex, hippocampus and white matter of AD subjects, compared with age-matched controls (Sengillo et al., 2013; Halliday et al., 2016; Miners et al., 2018; Schultz et al., 2018). Pericytes are highly susceptible to the toxic effects of Aβ, likely because of their ability to internalize the protein for attempted clearance across the BBB (Verbeek et al., 1997; Wilhelmus et al., 2007). This toxicity affects their overall survival as well as their structure. For example, in a mouse model of cerebral amyloid angiopathy affecting microvessels, not only were pericytes progressively lost with age, but the surviving pericytes were found to have unusually short processes, suggesting impairment in structural remodeling (Park et al., 2014). Since pericytes are also involved in removal of Aβ from the brain (Wilhelmus et al., 2007; Sagare et al., 2013; Candela et al., 2015), pericyte loss can initiate a “snowballing” effect to further increase Aβ burden, ultimately worsening microvascular injury in AD. This was exemplified through the accelerated Aβ accumulation, tau pathology, and worsened cognitive decline observed when cross-breeding a mouse model of AD pathology with a model of progressive pericyte deficiency (Sagare et al., 2013).

Aging is also a risk factor for small-vessel disease (SVD), which accounts for approximately 50% of all dementias including AD (Iadecola, 2013; Snyder et al., 2015). SVD is characterized by white matter hyperintensities (leukoaraiosis), lacunar infarcts, and microbleeds (Wardlaw et al., 2013). A recent study using a pericyte-deficient mouse model revealed pericyte loss as a mechanism of white matter degeneration (Montagne et al., 2018). In line with this idea, the walls of microvessels in human white matter become thinner with age, in part due to increased pericyte loss (Stewart et al., 1987). Genetic factors such as mutations in the NOTCH3, a cell surface receptor expressed by mural cells, cause a genetic form of SVD termed cerebral autosomal dominant arteriopathy with subcortical infarcts leukoencephalopathy (CADASIL). A recent study identified pericyte pathology as a primary source of vascular dysfunction in a mouse model of CADASIL (Ghosh et al., 2015).

Finally, it is worth mentioning that the description of pericyte pathology in the literature encompasses, besides cell death, a range of cellular abnormalities including detachment from the endothelium, migration, shape change, and in the case of brain injury, trauma and stroke, their phenotypic transformation. As noted by Kalaria, “it is likely that the brain vasculature is continually modified by growth and repair mechanisms in attempts to maintain perfusion during ageing and disease” (Kalaria, 1996). However, it is yet unknown if, and to what extent, pericytes can adapt in the face of vascular degeneration during aging and age-related diseases.

## Identifying Pericytes in the Adult Mouse Brain

**Figure 1:**
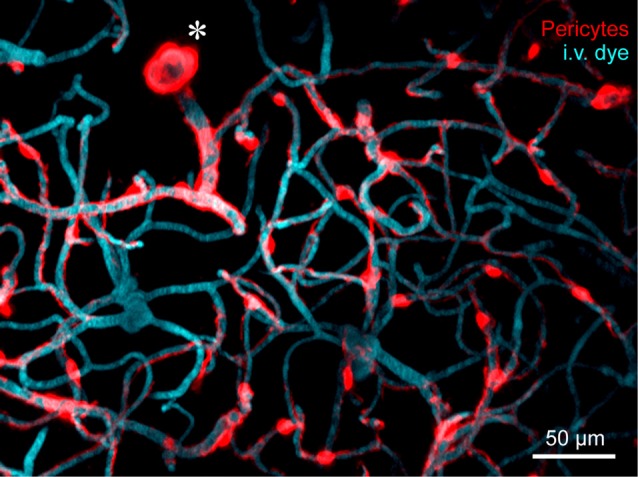
*In vivo* two-photon microscopy of adult mouse cerebrovasculature. High-resolution image of the capillary network surrounding a penetrating arteriole (*) in the mouse cortex. Mural cells are labeled red through expression of tdTomato driven under the control of the Myosin heavy chain 11 (Myh11) promoter. The vasculature is labeled with intravenously administered 2-MDa FITC-dextran, and pseudo-colored blue. Many inducible and constitutively active Cre drivers are suitable for imaging brain mural cells. For more information, see Hartmann et al. (2015a,b).

**Figure 2:**
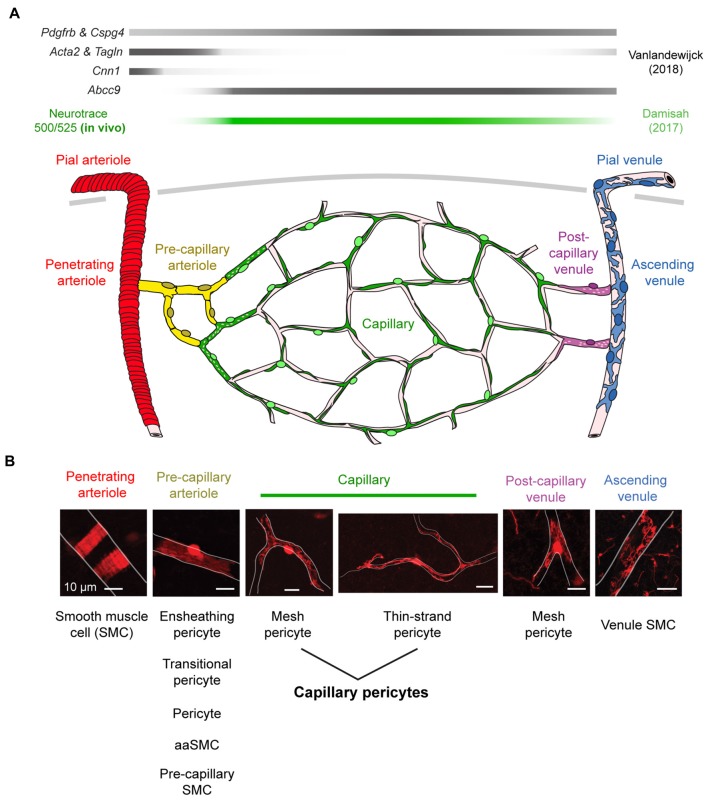
Mural cell heterogeneity in the adult mouse cerebrovascular network. **(A)** (Top) Mural cell gene expression in different zones of the microvasculature in cerebral cortex. Adapted from Vanlandewijck et al. (2018). Neurotrace 500/525 is specifically taken up by capillary pericytes, providing a cell-specific fluorescent label for *in vivo* imaging (Damisah et al., 2017). (Bottom) Schematic depicting mural cells in seven microvascular zones from pial arterioles to pial venule of cerebral cortex. **(B)** (Top) Representative images showing morphology of mural cells in five zones below the cortical surface. All scale bars are 10 μm, except the penetrating venule image, which is 50 μm. Adapted from Hartmann et al. (2015a) and Grant et al. (2017). (Bottom) The evolving nomenclature for each cell type. Note that naming of mural cells in the pre-capillary arteriole zone is not universally agreed upon and has become a source of controversy.

A key step in understanding the physiological roles of pericytes is the ability to visualize the cells in real time *in vivo*. Two-photon imaging now allows researchers to both observe and manipulate fluorescently-tagged pericytes in cerebrovascular networks of the intact mouse brain (Figure 1). However, despite the precision with which pericytes can be visualized, there remains ambiguity on what to call a “pericyte” (Attwell et al., 2016). It is therefore important to first clarify the different microvascular zones in cerebral cortex, and mural cell types that reside within these zones. We consider seven different microvascular zones that connect pial arterioles to pial venules in cerebral cortex, including: (1) pial arterioles, (2) penetrating arterioles, (3) pre-capillary arterioles, (4) capillaries, (5) post-capillary venules, (6) ascending venules, and (7) pial venules (Figure 2A). Along this arteriovenous route, there are genes that mural cells in all zones express at high levels, such as *Pdgfrb* (PDGFRβ) and *Cspg4* (NG2). However, recent single-cell transcriptomic data has also revealed zonation in gene expression (Vanlandewijck et al., 2018). At the junction between pre-capillary arterioles and capillaries, there is a sharp transition in expression profile that punctuates mural cells into two broad groups. Upstream to this transition, pial arteriole to pre-capillary arteriole zones highly express proteins related to contractile machinery, such as *Acta2* (α-smooth muscle actin; α-SMA)*, Tagln* (smooth muscle protein 22-α), and *Cnn1* (calponin 1). In contrast, downstream mural cells from capillary to venule zones are characterized by high expression of membrane transporters, such as *abcc9* (ATP binding cassette subfamily C member 9), consistent with roles in BBB transport. This may explain why pericytes at the capillary zone preferentially uptake the FluoroNissl dye Neurotrace 500/525 when applied to the brain surface (Damisah et al., 2017).

**Figure 3:**
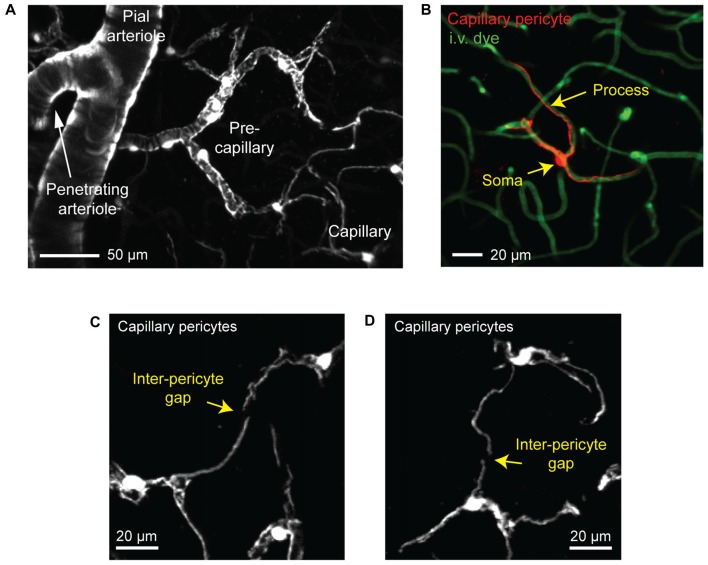
Visualizing capillary pericytes with *in vivo* two-photon imaging. **(A)** An *in vivo* image showing shift in mural cell appearance as a pial arteriole branches into an underlying penetrating arteriole, which then ramifies into the pre-capillary arteriole and capillary bed. The image was captured in the cortex of a Myh11-tdTomato mouse and is a maximal projection over 150 μm of cortical thickness. **(B)** *In vivo* image of an isolated capillary pericyte following intravenous injection of 2 MDa FITC-dextran to co-label the microvasculature. Image was captured from a NG2-tdTomato mouse. **(C,D)** Examples of the non-overlapping territories of adjacent capillary pericytes from a Myh11-tdTomato mouse. The location of gaps between two pericyte territories are denoted with arrows.

We next focus on the subsurface network of pre-capillary, capillary and post-capillary zones, where vessels are typically ≤12 μm. Mural cells of varying morphology line these zones (Figures 2B, 3A; Hartmann et al., 2015a; Grant et al., 2017). Most groups use a vessel branch order system to navigate the tortuous subsurface network, where branch order refers to the number of vessel bifurcations between the capillary of interest and a penetrating arteriole, which is denoted 0th branch order. Through detailed analyses of cortical vascular topology in mouse, we recently showed that the capillary zone can be reliably identified and distinguished from α-SMA expressing pre-capillary zone by examining capillaries that are more than four branch points from a penetrating arteriole (Grant et al., 2017).

We refer to pericytes of the capillary zone as “capillary pericytes.” These pericytes have conspicuous ovoid cell bodies that intermittently protrude from the vascular wall (Figures 2B, 3B). They extend thin, meandering processes that run along the vessel lumen for hundreds of micrometers (thin-strand pericyte). On the arteriolar pole of the capillary zone, capillary pericyte processes can adopt a mesh-like appearance that covers more of the abluminal surface area (mesh pericyte; Hartmann et al., 2015a; Grant et al., 2017). Critically, all capillary pericytes in the brain appear to express little to no α-SMA, and this has been verified by transcriptional analyses (Vanlandewijck et al., 2018).

Mural cells of the pre-capillary arterioles, a transitional zone between arteriole and capillary, are distinct from capillary pericytes. They are shorter in length, offer greater coverage of the endothelial surface, and like SMCs of pial and penetrating arterioles, are rich in α-SMA (Figure 2B). We have referred to these cells as “ensheathing pericytes,” i.e., a sub-type of pericyte, because they possess the hallmark protruding cell body of pericytes, and because α-SMA expression has historically been a marker for a subset of pericytes (Grant et al., 2017). Other groups have called these cells simply “pericytes” without distinguishing from capillary pericytes (Hall et al., 2014; Cai et al., 2018), transitional pericytes (Sweeney et al., 2016), pre-capillary SMCs (Hill et al., 2015), or aaSMCs (Vanlandewijck et al., 2018). The shifting nomenclature of these cells has been the root of recent controversy on pericyte roles in blood flow control (discussed further below), and a consensus on naming needs to be established.

The term “post-capillary venules” has been used in past literature, but whether this should represent a distinct microvascular zone with pericytes performing unique functions remains unclear. Pericytes of post-capillary venules have mesh-like processes, express little to no α-SMA, and are shorter than capillary pericytes (Hartmann et al., 2015a). There is evidence that venular pericytes of the cremaster muscle play a role in immune cell migration (Proebstl et al., 2012), but a similar role for venular pericytes in the brain remains to be examined.

Capillary pericytes are arranged along the capillary bed in a chain-like network, with the majority of the vasculature being contacted by their long cellular processes rather than cell bodies (Berthiaume et al., 2018). In fact, over 90% of the vasculature in the mouse cerebral cortex is contacted by pericyte processes, suggesting that pericyte-endothelial signaling may occur primarily through these processes (Underly et al., 2017). Interestingly, each pericyte occupies a defined territory that does not overlap with the territories of neighboring pericytes (Hill et al., 2015; Berthiaume et al., 2018). Pericyte territories are so precisely arranged that it is usually difficult to determine where one pericyte ends and the next begins, but for the occasional gap between processes of neighboring cells (Figures 3C,D).

## Pericyte Remodeling in the Adult Brain

**Figure 4:**
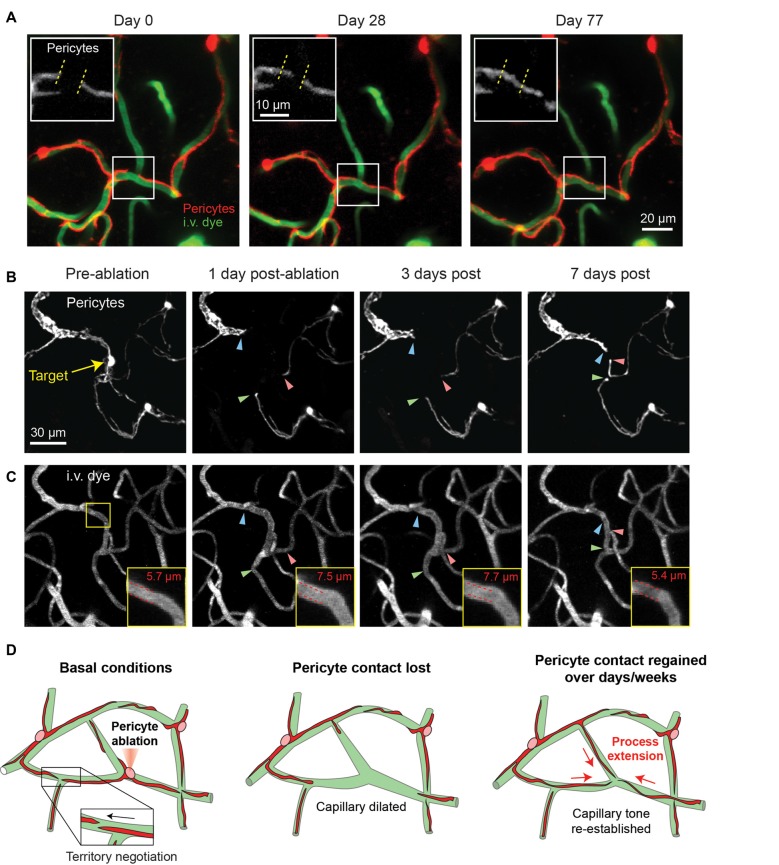
Pericyte structural remodeling captured with chronic *in vivo* 2-photon imaging. **(A)** An example of the structural dynamics of adjacent pericytes under basal conditions. Inset shows the extension of a pericyte process beyond its territory at Day 0, and the corresponding retraction of a neighboring pericyte process. Images are from a Myh11-tdTomato mouse. Adapted from Berthiaume et al. (2018). **(B)** Two-photon laser ablation of a single pericyte results in the robust extension of immediately adjacent pericyte processes into the vacated territory over 7 days. The image shows the extension of two thin-strand pericytes (green and red arrowheads) and one mesh pericyte (blue arrowhead). Images are from a Myh11-tdTomato mouse. **(C)** Images of the vasculature, labeled by 2 MDa FITC-dextran, at the site of pericyte ablation (same region as panel **B**). Inset shows an increased capillary diameter in the vessel segment lacking pericyte coverage, which returns to baseline diameter once pericyte contact is regained suggesting vascular tone. **(D)** Schematic of pericyte structural remodeling under basal conditions and following acute pericyte ablation.

In a recent long-term *in vivo* imaging study, we showed that capillary pericytes under basal conditions negotiate their individual territories with neighboring pericytes through slight extensions and retractions of their terminal processes (Berthiaume et al., 2018). When inter-pericyte gaps were visible, the push-pull interplay between adjacent pericytes could be observed, suggesting that pericyte domains might be maintained throughout adulthood by repulsive pericyte-pericyte interactions (Figure 4A). The extent to which neighboring pericytes make direct contact with each other remains unclear. However, recent studies in the retina have suggested gap junction communication between neighboring pericytes involved in conductive vasomotor constrictions (Ivanova et al., 2017).

While the basal changes in pericyte structure we observed were small, it nonetheless raised the question of whether mature brain pericytes are structurally plastic, and if this plasticity could be further induced. To examine this idea, we ablated single capillary pericytes using precise two-photon laser irradiation. This enabled the specific and immediate deletion of individual cells anywhere within the cortical capillary network. In the days to weeks following ablation, the processes of neighboring pericytes extended into the territories uncovered by the ablated pericyte with ~10-fold greater speed and magnitude than dynamics observed under basal conditions (Figures 4B,C). Process extension occurred while the pericyte somata remained firmly affixed, indicating no overt cell migration or proliferation. New cytoplasmic material was added to the growing pericyte process, enabling contact with hundreds of micrometers of extra capillary length. Importantly, this work demonstrated that pericytes can remodel their shape and grow in size in the adult brain and are inherently programed to maintain coverage of the endothelium. Yet, the mechanism appears imperfect, as the need to increase cytoplasmic volume suggests a limit to growth, and many days were required to regain endothelial coverage. Whether this reparative process can be pharmacologically enhanced to promote continued pericyte-endothelial contact in the adult brain will be important to explore.

A logical molecular target to modulate pericyte structural remodeling is PDGF-B/PDGFR-β signaling. PDGF-B delivery is neuroprotective in stroke models and PDGFR-β expression in pericytes increases progressively following cerebral ischemia in the adult brain (Arimura et al., 2012), likely reflecting a reparative response. Also relating to PDGF-B/PDGFR-β signaling, Lebrin et al. (2010) showed that the drug thalidomide reduced bleeding in patients with hereditary hemorrhagic telangiectasia (HHT), a disease involving vascular malformations and bleeds in multiple organs. Using a mouse model of HHT, the benefit of thalidomide was shown to be partly due to the enhancement of endothelial PDGF-B expression leading to increase of pericyte recruitment/coverage. Similarly, thalidomide was reported to promote PDGF-B/PDGFR-β signaling, increase pericyte coverage, and reduce bleeding in a mouse model of brain arteriovenous malformation (bAVM; Zhu et al., 2018), a cerebrovascular disease with marked pericyte loss in humans (Winkler et al., 2018). Whether enhanced pericyte structural remodeling is a salutary effect of thalidomide needs further investigation. However, it seems likely in light of recent work reporting increased pericyte process growth following treatment with PDGF-BB in a mouse model of epilepsy (Arango-Lievano et al., 2018).

A second molecular interaction that may be at play in adult pericyte structural remodeling is Eph-ephrin signaling. It is currently unknown whether this bidirectional signaling pathway acts basally as a repulsion and/or cell-spreading signal for defining pericyte territories in the adult brain. If so, pericyte loss in the adult brain could disrupt pericyte-to-pericyte Eph-ephrin repulsive signaling, essentially disinhibiting the growth of pericyte processes. This, in conjunction with the expression of other directional growth cues (i.e., PDGF-B), could help explain our observation following single pericyte ablation, where neighboring processes robustly spread into vacated territories until contact with another pericyte is made.

## Consequences of Pericyte Loss in the Adult Brain

The ability to selectively remove pericytes from an existing vascular network and then track recovery over time provided insight into the physiological consequence of pericyte loss in the adult brain. We examined three modes of pericyte function that are well-established from studies of developing organisms, including regulation of: (1) capillary network structure, (2) BBB integrity, and (3) capillary lumen diameter.

First, we found that single pericyte loss in mature vascular networks had no overt effect on capillary structure. That is, no formation or elimination of new capillary branches was observed in regions without pericyte coverage. This was an unexpected finding, as pericyte loss could conceivably lead to capillary pruning due to loss of endothelial support, or aberrant angiogenesis by alleviating the suppression of endothelial proliferation. Thus, capillaries in the adult brain can be structurally stable with transient loss of pericyte coverage *in vivo*. It will be important to determine if and how aging or cerebrovascular disease makes the vasculature more vulnerable to pericyte loss. Second, we were unable to detect acute BBB leakage from uncovered capillary segments following single pericyte ablation. This was also surprising, as pericyte loss in the developing brain is strongly associated with increased endothelial transcytosis. Even with the use of small molecular weight dyes (1 kDa), which have been previously shown to permeate through a trans-cellular route (Armulik et al., 2010), we found no evidence of plasma extravasation at capillary regions lacking pericyte coverage. In line with this lack of BBB leakage, a recent study using an inducible diphtheria toxin strategy to acutely kill mural cells, including pericytes, in adult mice reported intact blood-retinal and blood-brain barriers, despite vascular leakage in peripheral tissues (Park et al., 2017). Furthermore, mouse models of mild pericyte deficiency retaining approximately 70% of normal pericyte coverage in the adult cerebral neocortex do not present BBB leakage, while a drop to 45% coverage results in a modest BBB leak (Armulik et al., 2010). Collectively, these findings suggest that BBB integrity in the adult brain can be resilient to some degree of pericyte loss.

One consistent alteration we did observe after single pericyte ablation, however, was the sustained dilation of the capillary lumen in regions lacking pericyte contact (Figures 4B–D). This dilation persisted until pericyte processes grew back into the exposed territory, at which point capillary diameter returned to basal levels. This finding was mirrored in a study examining the dynamic loss and gain of mural cell coverage induced by seizure activity (Arango-Lievano et al., 2018). This implies that pericytes at the capillary level exert a steady-state vascular tone that may behave like a tension clamp on the endothelial tube either mechanically or through constant molecular signaling with the endothelium.

The idea that pericytes regulate blood flow has persisted through the literature since their discovery by Rouget in the late 1800s (Rouget, 1873), but has remained a controversial topic to this day (Attwell et al., 2016). The majority of *in vivo* imaging studies to date have focused on whether pericytes are involved in the second-to-second diameter changes need for blood flow control during neurovascular coupling. This issue is challenging to address because constriction or relaxation of upstream arterioles will influence blood flow into downstream capillaries, making it very difficult to attribute changes in capillary blood flow to autonomous action by capillary pericytes *in vivo*. Many have circumvented this issue by studying pericyte contractility *ex vivo* in a variety of CNS tissues, such as cortical/cerebellar slices and isolated retina. These studies agree that pericytes of capillary and pre-capillary zones can respond to electrical (Peppiatt et al., 2006; Mishra et al., 2016; Ivanova et al., 2017), pharmacological (Peppiatt et al., 2006; Fernández-Klett et al., 2010; Hall et al., 2014) and neural stimuli (Hall et al., 2014; Biesecker et al., 2016; Mapelli et al., 2017). However, some caution needs to be taken for interpretation as *ex vivo* systems exhibit capillary diameter changes on much longer timescales (2 min), with relevance to neurovascular coupling less certain. Further, mixing pericytes functions across different tissues (brain vs. retina) is problematic because pericytes functions and vascular architecture differ. In fact, a recent study using modified tissue fixation procedures suggests that retinal capillary pericytes express high α-SMA (Alarcon-Martinez et al., 2018). However, measurements of *acta2* transcripts (Vanlandewijck et al., 2018) and use of *acta2* Cre-recombinase drivers combined with potent reporters (Hill et al., 2015) suggest little to no α-SMA expression in brain capillary pericytes.

*In vivo* imaging studies agree that mural cells of pre-capillary arterioles support rapid diameter changes required for neurovascular coupling, though nomenclature for these cells differ between labs (Hall et al., 2014; Hill et al., 2015). In fact, dilatory and constrictive responses may initiate at pre-capillary arterioles and then conduct upstream to penetrating arterioles (Cai et al., 2018). However, the role of capillary pericytes in dynamic control of capillary diameter remains debated. Some groups have reported no capillary diameter changes with optogenetic stimulation (Hill et al., 2015), neural activity (Fernández-Klett et al., 2010; Hill et al., 2015; Wei et al., 2016) or ischemia (Hill et al., 2015), while other groups have observed diameter changes with neural activity (Hall et al., 2014; Kisler et al., 2017).

One way to reconcile these data is that capillary pericytes are contractile, but much less so than their counterparts on upstream arterioles. Capillary pericytes may be required for establishing basal, long-term equilibrium and optimum flow through the capillary bed, whereas upstream mural cells are responsible for initiating rapid moment-to-moment changes in blood flow. A constant, steady-state tone imparted by capillary pericytes is a less studied aspect of cerebral blood flow control. However, it is critical for brain function as all blood entering the brain must percolate through the dense, pericyte-covered capillary bed, irrespective of local neuronal activity. Given that red blood cells are larger than the average diameter of the capillary lumen and must deform to pass through (Secomb, 1991), even small alterations in basal capillary diameter will have a significant effect on capillary transit time and oxygen availability (Jespersen and Østergaard, 2012). An estimated 40% of total cerebrovascular resistance exists at the level of penetrating arterioles, capillaries, and venules Iadecola (2017), and modeling studies suggest that the capillaries confer a sizable proportion of this resistance (Gould et al., 2017).

Little is known about how age or disease-dependent pericyte loss affects basal capillary diameter, blood flow and oxygen delivery. One recent study examined young *pdgfrb*^+/–^ mice, which exhibit a moderate loss of pericyte coverage (from 77% coverage in wild-type to ~55% in knockouts), and showed impaired basal tissue oxygen supply using a novel oxygen-sensitive imaging probe (Kisler et al., 2017), but no change in basal capillary diameter. However, in adult *pdgfrb*^ret/ret^ mice, which exhibit more severe loss of pericyte coverage (from ~90% coverage in wild-type to ~25% in knockouts), capillary diameters became significantly larger, consistent with the idea of altered basal tone with sufficient pericyte loss (Armulik et al., 2010). Further, a study of human bAVMs showed increased blood flow within the AVM nidus (shorter mean transit time), as measured by pre-operative angiograms, when pericyte coverage dropped below 40%, suggestive of capillary dilation. Like capillary constriction and impedance of flow (Yemisci et al., 2009; Hall et al., 2014), capillary dilation can disrupt normal blood flow rate and blood cell distribution within the microvascular network, and this has the potential to alter oxygen delivery to tissues (Schmid et al., 2015).

## Outlook

The structural remodeling of pericytes in the adult mouse brain may be essential for maintenance of cerebrovascular health and needs to be broadly explored in models of cerebrovascular disease. This task is facilitated by long-term *in vivo* imaging methods that allow quantification of pericyte growth, coverage, and capillary flow over time. Among the critical next steps are the need to examine the physiological consequence of pericyte ablation at the *adult* stage using either precise optical methods (Hill et al., 2017; Berthiaume et al., 2018), or novel pericyte-specific Cre drivers to delete or modify capillary pericytes (Zlokovic et al., 2015; Park et al., 2017). While many studies have used mice with congenital deficiency in pericyte-endothelial signaling, very little is known about the impact of pericyte loss in models where pericytes develop normally at the beginning. Embedded in this broader issue are intriguing questions of whether all pericytes are functionally homogeneous, or whether pericytes in some microvascular zones lack the capacity to remodel and are more vulnerable to vascular disease. More work is also needed to determine the threshold for pericyte loss that surpasses the compensatory ability of pericyte process growth, which presumably contributes to the development of neurovascular pathologies in the adult and aging brain. The effects of age and cerebrovascular disease on pericyte structural remodeling will be important areas of future inquiry as well. Lastly, the molecular signaling that governs pericyte remodeling in adulthood requires further study, with PDGF-B/PDGFR-β and Eph-ephrin signaling as logical targets for pharmacological or genetic manipulation in the adult brain.

## Author Contributions

A-AB wrote the review with feedback from MM, NB and AS.

## Conflict of Interest Statement

The authors declare that the research was conducted in the absence of any commercial or financial relationships that could be construed as a potential conflict of interest.
